# Effects of Combined α-Amylase and Endo-Xylanase Treatments on the Properties of Fresh and Frozen Doughs and Final Breads

**DOI:** 10.3390/polym12061349

**Published:** 2020-06-15

**Authors:** Hye-Jin Kim, Sang-Ho Yoo

**Affiliations:** Department of Food Science and Biotechnology, and Carbohydrate Bioproduct Research Center, Sejong University, 209 Neungdong-ro, Gwangjin-gu, Seoul 05006, Korea; chossa613@naver.com

**Keywords:** bread baking, carbohydrate, enzyme, loaf volume, crumb softness

## Abstract

Frozen bread doughs usually exhibit less bread volume and poor texture due to dough weakening as well as reduced yeast viability. The objectives of this study were to improve the textural properties of frozen bread dough by applying carbohydrate-active enzymes, α-amylase and endo-xylanase. Each enzyme was applied to dough formulation at 20 (748 and 3.5 units, respectively) and 100 ppm levels of flour, and their combined treatments were also applied. Enzyme-treated doughs were kept frozen at −20 °C for 2 weeks, and then baked following the official American Association of Cereal Chemists (AACC) method. A texture profile analysis of oven-baked breads was performed at 25 °C after a 5-day storage period. α-Amylase treatment at a 100 ppm level increased the specific bread volume by 24.5% and 21.9% when compared to untreated fresh and frozen bread doughs, respectively, and decreased crumb hardness by 63.4% and 58.3%; endo-xylanase (100 ppm) also decreased crumb hardness by 56.9% and 26.9%. The combined use of α-amylase and endo-xylanase retarded bread hardening synergistically after a 5-day storage period.

## 1. Introduction

The bakery industry has increasingly utilized the applications of freezing technology, and there are many papers on the storage of frozen doughs and the influence of such processes on the quality of the final product. The growing interest of the market toward frozen bakery goods is due to the economic advantage of a centralized manufacturing and distribution process as well as easier standardization of product quality. These products do not require specialized workers and raise the possibility of making “fresh” bread available at any time of the day [1]. Frozen dough has several baking problems such as poor bread volume and lower texture properties that have been ascribed to dough weakening and reduced yeast viability. The gradual loss of dough strength during frozen storage has been attributed to the reduction in gluten crosslinking caused by ice crystallization, the release of reducing substances from yeast, and the water redistribution provoked by a modification in the water binding capacity of dough constituents [2,3,4]. These phenomena may produce a loss in the gas retention capacity during fermentation, reflected by lower bread volume and an increase in fermentation time [4]. Thus, to obtain a product from frozen dough with a quality comparable to freshly made bread is a complex problem since the final structure is modified by several parameters [5]. To improve frozen dough, several technical modifications have been introduced in recent years. These include (1) the isolation of freeze-resistant yeasts [6]; the (2) addition of improvers such as emulsifiers and water-binding agents, e.g., hydrocolloids to stabilize the dough network; (3) the addition of wheat proteins to increase shelf life [7]; (4) the modification of dough composition [8]; (5) the use of heat stable enzymes to shorten fermentation time [9]; (6) the optimization of mixing, freezing and freeze–thaw cycles [10,11].

Enzymatic processes among various modification methods can effectively modify the structures of wheat polysaccharides, such as starch and non-starch polysaccharides, and the main components of cereals, which usually brings positive effects on dough and bread properties. The α-amylase (Aamyl) (EC 3.2.1.1) hydrolyses α-1,4-glycosidic linkages of starch and produces maltose, maltotriose, maltooligosaccharides, branched oligosaccharides, and low molecular weight α-limit dextrins. The added amylases increase the level of fermentable and reduce sugars in flour and dough, thus promoting yeast fermentation and the formation of Maillard reaction products, which in their turn, intensify bread flavor and crust color [12,13]. The molecular structure changes that occur in bread during storage which are responsible for the firming and staling phenomenon are quite complex and involve multiple constituents and mechanisms [14]. It is generally accepted that after the initial cooling process, the retrogradation/recrystallisation of starch, specifically of amylopectin branch chains in amylopectin, plays a major role in bread firming [12,14]. Starch retrogradation occurs in two different time-dependent stages [15,16]: amylose crystallization takes place after leaching out from starch granule in the short-term phase, while recrystallization through the outer branches of amylopectin is considerably slower than amylose retrogradation. Meanwhile, certain bacterial Aamyls have been known to decrease the rate of bread firming [17]. Endo-β-1,4-xylanases (EC 3.2.1.8.), also referred to as endo-xylanases (Exyl) or xylanases, have found widespread use in breadmaking applications since 1970s. Indeed, it has been well reported that the Exyls most suited for breadmaking are those active on the water-unextractable fraction and poorly active on the water-extractable fraction, solubilizing the insoluble arabinoxylan to give high molecular weight solubilized arabinoxylans and hence leading to a net loss of water holding capacity and an increased viscosity [18,19]. This action not only removes the insoluble arabinoxylan which interferes with the formation of the gluten network in dough, but also increases the stability of the dough system due to the increased viscosity. This in turn yields a more stable and flexible dough, which is easier to handle and gives improved oven spring, a larger loaf volume, improved crumb structure (fine crumbs and thin gas cell walls) and hence also a softer crumb [18]. Despite factual acceptance of Exyl as a means of improving dough and bread characteristics, their mode of action is still the subject of debate [20,21]. The objectives of this study were to understand the effects of Aamyl and Exyl treatment on the bread quality of frozen dough, which were compared with that of fresh dough.

## 2. Materials and Methods

### 2.1. Materials

Commercial strong wheat flour was used for all experiments and was provided by Samyang Co. (Seoul, Korea). Enzymes included endo-β-1,4-xylanase (Pentopan 500 BG, Novozyme, Bagsvaerd, Denmark), and fungal α-amylase (Fungamyl 2500 SG, Novozyme, Bagsvaerd, Denmark). Baker’s yeast was obtained from Societe Industrielle Lesaffre (Marcq-en-Baroeul, France). Salt and sugar were the products of CJ Cheiljedang Co. (Seoul, Korea). Shortening was obtained from Ottogi Co. (Seoul, Korea), and L-ascorbic acid was from Sigma-Aldrich Chemical Co. (St. Louis, MO, USA).

### 2.2. Activity Determination of Endo-β-1,4-Xylanase and α-Amylase

Endo-β-1,4-xylanase and α-amylase activities were assayed by measuring the release of reducing sugar that were produced from beechwood xylan (Megazyme, Sydney, Australia) and soluble starch (Yakuri Pure Chemicals Co., Kyoto, Japan) hydrolysis, respectively. The endo-β-1,4-xylanase assay was carried out in 50 mM sodium acetate (pH 7.0) with 1% (w/v) beechwood xylan at 50 °C for 10 min. The α-amylase assay was performed in 50 mM sodium acetate (pH 5.0) with 1% (w/v) soluble starch at 50 °C for 10 min. The reactions were stopped by 500 µL of dinitrosalicylic acid (DNS) reagent, and then the reaction tubes were soaked in boiling water for 5 min. The absorbance of the solutions was measured at 575 nm. One unit of endo-β-1,4-xylanase and α-amylase was defined as the amount of enzyme required to release 1 μmol of reducing end per min. The activities of them were converted to the units of ppm because the enzymes were supplied as a form of powder. One part per million (ppm) of endo-β-1,4-xylanase and α-amylase was equivalent to 0.19 units and 37.4 units, respectively.

### 2.3. Bread-Making Procedure

Carbohydrate-active enzymes, such as endo-β-1,4-xylanase and α-amylase, were applied for bread-making. The bread dough formula consisted of wheat flour (300 g), dehydrated yeast (6.0 g), salt (4.5 g), sugar (18 g), shortening (9 g), ascorbic acid (200 ppm), and water (175.5 mL). The desired moisture content for dough mixing was determined based on the result of the Mixolab analysis (Chopin Technologies, Villeneuvela Garenne, France). The parameters obtained from the recorded curve were the amount of water required for the dough to produce a torque of 1.1 ± 0.07 Nm. The ingredients were mixed for 3.5 min at 25 °C in a 500 g pin mixer (National Manufacturing Co., Inc., Piscataway, NJ, USA). The dough was then divided into 150 g pieces. This fresh dough was fermented immediately while frozen dough was baked after a 2-week storage at −20 °C (without a given thawing time). These fresh and frozen doughs were fermented at 35 °C and 90% relative humidity (RH) for 180 min, and the dough samples were punched two times in the middle of fermentation at 105 and 155 min, respectively, and finally at 180 min once more just after fermentation. After the molding process of the doughs, they were proofed for 62 min and baked at 210 °C for 24 min in an oven (Daeyung Co., Seoul, Korea).

### 2.4. Extraction and Analysis of Water-Soluble Arabinoxylan in Bread Crumb

After the bread was baked in the oven and air-cooled on the lab table, the lyophilized and ground bread crumb sample was prepared, and it was suspended in deionized water (crumb:water = 1:5, w/v). Water-soluble solids were extracted from the resulting slurry at 5 °C for 1 h [5]. After centrifugation (5000× *g*, 4 °C and 10 min), the soluble extract was boiled and treated with thermostable α-amylase for 6 h. Once starch liquefaction was completed, the reaction mixture was cooled to 60 °C, and the protease treatment was pursued at 60 °C for another 6 h to remove proteins. After the enzyme-treated samples were boiled for 20 min, its pH was adjusted to 4.3 by adding 200 mM of sodium acetate buffer containing 1% (w/v) sodium azide. By adding amyloglucosidase to the sample solution, starch fragments were completely hydrolyzed at 60 °C for 24 h, which was centrifuged at 10,000× *g* for 10 min. To the supernatant, five volumes of ethanol were added and held at 4 °C overnight. The precipitate was washed twice with 80% ethanol and was allowed to dry in the air until no solvent could be detected by odor. This purified water-soluble arabinoxylan (WS-AX) was dissolved in water at 60 °C for 2 h, filtered through a polypropylene membrane filter (0.45 µm pore size), and then injected (100 µL) with 10 mg/mL to the HPLC system for the analysis of structural changes in the WS-AX component.

### 2.5. Analysis of Bread Loaf Volume and Crust Color

The loaf volume of the bread sample was determined with a Volscan Profiler (Stable Microsystems, Godalming, Surrey, UK). The crust color of the bread was determined according to the AACC Approved Method 14–22 (AACC International, 2000) using the Minolta chromameter (CR-300, Minolta Camera Co. Ltd., Osaka, Japan). The chromameter was calibrated with a Minolta calibration plate, and the C standard illuminant was used. Data were expressed as lightness/darkness (L*), redness/greenness (a*), and yellowness/blueness (b*). The crust color was measured on the top of each bread sample in triplicate.

### 2.6. Texture Profile Analysis of Bread Crumb

Textural characteristics of bread sample were examined just after cooling the bread and at 5 days after baking by the texture analyzer (TMS-Pro, Mecmesin Ltd., West Sussex, UK) equipped with a 25-N load cell. The average values determined from three different loaves were reported as data points. At least two slices of bread (20 mm thickness each) cut from each loaf center were applied for this analysis. The bread slices were compressed up to 14 mm (equivalent to 70% of slice height) from the cross section of bread crumb with a 25 mm diameter cylindrical probe at a test speed of 60 mm/min. The crumb firmness and elasticity were evaluated by applying compression two times in each cycle.

### 2.7. Statistical Analysis

ANOVA and Tukey’s honestly significant difference tests were performed using Sigmaplot software package 13.0 (Systat Software Inc., San Jose, CA, USA).

## 3. Results and Discussion

### 3.1. Molecular Weight Distribution of Water-Soluble Arabinoxylan (WS-AX) Extracted from the Bread Crumb

**Figure 1:**
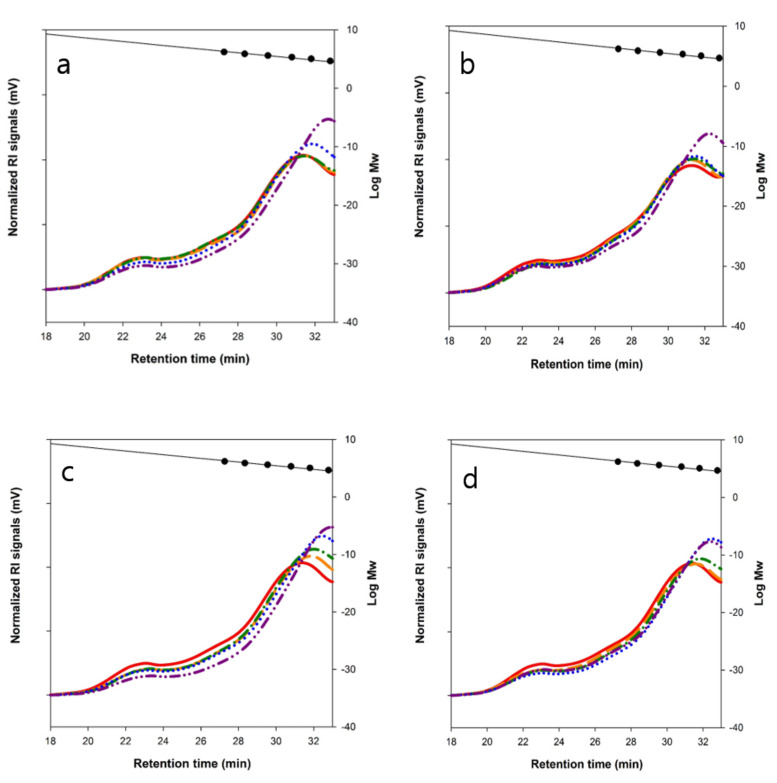
High-performance size-exclusion chromatography (HPSEC) analysis of water-soluble arabinoxylan (WS-AX) extracted from fresh and frozen bread doughs treated with carbohydrate hydrolyzing enzymes. A, FSDB treated with Aamyl (A) or Exyl (E); B, FZDB with Aamyl (A) or Exyl (E). In panels (**a**) and (**b**), untreated control, **——**; A20, **———**; A100, **– ∙– ∙–**; E20, **∙∙∙∙∙**; E100, **– ∙∙– ∙∙–**. C, FSDB treated with Aamyl (A) and Exyl (E); D, FZDB treated with Aamyl (A) and Exyl (E). In panels (**c**) and (**d**), untreated control, **——**; A20E20, **———**; A100E20, **– ∙– ∙–**; A20E100, **∙∙∙∙∙**; A100E100, **– ∙∙– ∙∙–**. The values following the letters of A and E mean the level of enzyme treatment in ppm. Molecular size makers spotted inside the chromatogram from the left to the right are 1600, 788, 400, 212, 112, and 47.3 kDa.

To improve the quality of frozen bread dough (FZDB; dough that was frozen at −20 °C for 2 weeks), the enzymes, α-amylase (Aamyl) and endo-xylanase (Exyl), were formulated in the dough making. Water-soluble arabinoxylans (WS-AXs) were extracted from the enzyme-treated bread crumb, and their relative Mw distributions were analyzed by HPSEC. As the result of increasing the dose level of Exyl activity in both the fresh dough bread (FSDB) and the FZDB, the Mw of WS-AX noticeably decreased (Figure 1a,b). The increase in the dose level of Aamyl activity in both FSDB and FZDB did not significantly change the AX structure from the control as expected from the substrate specificity of Aamyl (Figure 1). Even in the dough samples treated with both Aamyl and Exyl, it was clearly shown that Aamyl did not affect the size of WS-AX (Figure 1c,d).

**Figure 2:**
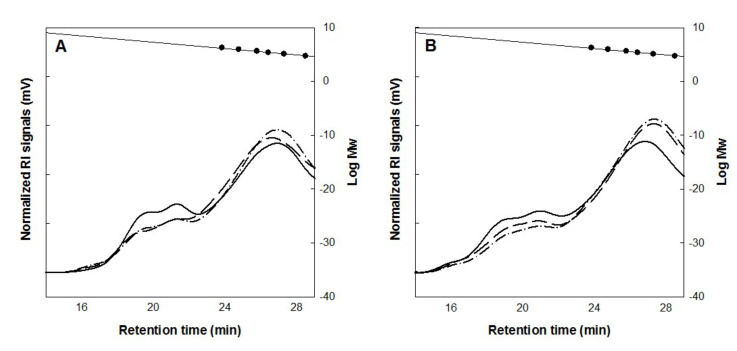
High-performance size-exclusion chromatography (HPSEC) analysis of water-soluble arabinoxylan (WS-AX) extracted from fresh and frozen bread doughs. Fresh (**A**) and frozen bread dough (**B**) formulated with Exyl. After mixing, ——; after proofing, ——; after baking, –∙–∙–. Molecular size makers spotted inside the chromatogram from the left to the right are 1600, 788, 400, 212, 112, and 47.3 kDa.

Even though the action of Exyl might not be significant during the freezing storage period, the enzyme remained stable and could be well activated by thawing, and thus the reactivated Exyl probably hydrolyzed AX during mixing, fermentation, and baking of the FZDB (Figure 2). In comparison to FSDB, the FZDB clearly displayed less hydrolysis of WS-AX but still significant decrease in Mw of WS-AX was observed. It could be expected that reactivation of the enzymes in frozen dough might take an induction period and low temperature storage could inactivate a certain part of the enzyme, which delayed the hydrolysis of the AX structure. It was previously reported that the Mw reduction in WS-AX led to the decrease in dough viscosity [22] and AX-based oligosaccharide products had a health-beneficial effect as a prebiotic [23].

### 3.2. Effects of Carbohydrate-Active Enzyme Treatment on Wheat Bread Quality

**Figure 3:**
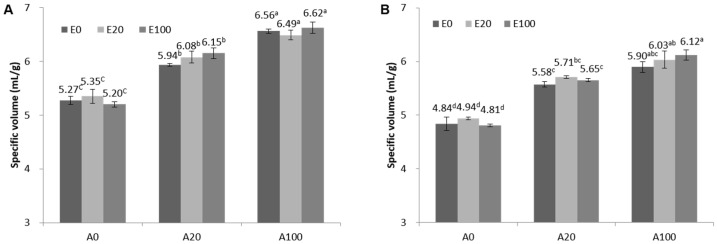
Specific loaf volume profiles of fresh and frozen bread doughs formulated with Aamyl and Exyl. (**A**) FSDB; (**B**) FZDB. The values with different letters (^a,b,c,d^) are significantly different at *p* < 0.05 within a clustered group of both panels A and B.

To determine the effects of Aamyl and Exyl, which are active enzymes in starch and cell wall polysaccharides, respectively, the quality of breads made from the FSD and FZD was evaluated by analyzing the quality parameters such as loaf volume, textural properties, and crumb color of the breads. The loaf volume of bread affects consumer preference on product purchase as one of the important physical properties of bread. The specific volume of untreated FZDB (4.84 mL/g) was smaller by 8.4% than that of FSDB (5.27 mL/g) (Figure 3). Not only between the untreated controls, but the comparison between enzyme-treated FSDB and FZDB also showed that the specific volume of FZDB was consistently lower than that of FSDB at the same enzyme treating levels. This smaller specific volume of FZDB might be due to its lower gas retention capability through a weaker gluten network by ice crystals formed in the freezing process. Moreover, the viability of yeast cells in thawed dough was negatively affected by freezing storage, and less CO2 production during baking resulted in incomplete pore swelling and a reduction in the specific volume of bread when compared to fresh baked bread [24,25].

In this study, we also investigated the effect of enzyme treatment on the specific volume of FSDB and FZDB. As a result, both in FSDB and FZDB, Exyl treatment did not significantly change it from the control as expected from the previous study [26]. When 20 and 100 ppm (flour weight basis) of Aamyl were applied in the dough formulation, the increase in specific volume was observed by 12.7 and 24.5%, respectively, in FSDB (Figure 3). Meanwhile, 15.3 and 21.9% of volume increases were obtained, respectively, by adding 20 and 100 ppm of Aamyl from the FZDB. When Aamyl and Exyl were treated together, a volume increase by Aamyl was still clearly shown, but Exyl effect on the volume was not detectable at all. Accumulation of fermentable reducing sugars by Aamyl action accelerated CO_2_ production by yeast, which resulted in larger air cells by high gas pressure in the dough. In the case of Exyl-treated dough, it was generally reported that this enzyme treatment also improved the bread volume but no significant change in the volume was observed in our study. This inconsistency might be due to the variation of the amount and properties of pentosans depending on wheat cultivars and thus a different action pattern of Exyl on a wide range of these substrates [27]. In addition, it was reported that a decrease in pentosan content or destruction of pentosan did not affect bread volume as well [26].

### 3.3. Texture Profile Analysis of Enzyme-Treated Bread Crumbs

Textural analysis of food materials is an important technical tool for evaluating consumer acceptance on the processed products. The parameters obtained from food texture analysis seems to be indirectly representing most of the critical attributes affecting the organoleptic properties. In case of oven-baked goods, its crust and crumb are generally recognized as the objects for the texture analysis. Fresh oven-baked bread displays a firm crust while its crumb exists as a porous structure induced by CO_2_ gas released by yeast fermentation, and these final air cells in the crumb are mainly interconnected by a gluten–starch network [28].

When the breads were treated with carbo-active enzymes, the hardness and chewiness of FSDB decreased to 1.24 N and 0.96 J with a high dose of Aamyl (A100), respectively, and decreased to 1.46 N and 1.17 J with a high dose of Exyl (E100) as well, from 3.39 N and 2.60 J of these parameters of control bread. When combined treatment of a high dose of both Aamyl and Exyl (A100E100) was applied to fresh bread dough formulation, these parameters decreased to 1.19 N and 0.93 J, respectively, of which the result showed somewhat decreasing effects on both hardness and chewiness but displayed no statistically significant differences (Table 1). Furthermore, Aamyl (A100) seemed to act more effectively than Exyl (E100) did in relation to the textural change in the breads. The hardness of the crumb is closely related to the structure of gas cell walls, especially, which is affected by the size and number of gas cells. It was reported that the larger size of gas cells by adding Aamyl resulted in bread with a softer crumb [26,29]. It was also previously reported that a small change in the loaf volume of bread might cause a large difference in crumb hardness [30]. Indeed, the crumb hardness of untreated control FSDB was almost three times greater than that of FSDB with high doses of Aamyl and Exyl (A100E100) whereas the volume difference between them was only 25% in our present study. Aamyl was known to partially hydrolyze amylopectin chains in starch molecules, and thus decreased the degree of its recrystallization and prevented water molecule fixation inside retrograded starch structures [31,32,33].

In this experiment, the texture of baked FSDB and FZDB was analyzed after 5 days of storage at room temperature. From the control breads, the hardness of FSDB and FZDB were measured to be 7.22 and 9.59 N, respectively, which showed that the hardness of FZDB increased more rapidly than that of FSDB along with storage time (Table 2). Water molecule migration, and ice crystal formation and recrystallization during the freezing-storage period of FZDB might cause the starch component to restructure, in turn leading to the bread hardening process due to the changes in gelatinization and retrogradation patterns of starch molecules [34]. Along with the retrograding process, water molecules could move into an amylopectin crystal structure and resided inside. These crystalline hydrate water molecules might not have the capability of network formation, which resulted in disrupted flexibility of the gluten network and increased crumb hardness [14]. As a result, the degree of retrogradation substantially increased as the storage time was extended in the freezing condition [3,35,36]. Water-unextractable arabinoxylan (WU-AX) is one of the important polymeric materials maintaining the structural integrity of cereal endosperm cells. When this cell wall component is disrupted by one of hemicellulose-type enzymes, xylanase, the crumb hardness is subjected to decrease by its plasticizing effect on the gluten–starch network in the breads [27,37]. The antistaling effect of xylanase on breads was observed by DSC analysis previously, which suggested that xyalanase action partially prevents the retrogradation of the starch component in the beard crumb [14,38,39].

### 3.4. Effects of Enzyme Treatment on the Color of Bread Crust

The color of bread crust is one of the important quality factors affecting consumer preference expected from bread products. In general, bread crust is characterized as low moisture and dark brown in color. The typical bread color on the crust is generated by chemical reactions such as the Maillard reaction and caramelization. As the dosage level of Aamyl increased in the bread dough formulation, reducing sugar formation was accelerated and the released sugars were utilized for the Maillard reaction. The resulting outcome displayed low L* values and led to a much darker crust formation. The crust color between light and dark brown was preferred by most consumers, and the flavor and taste of baked bread could be improved as the result of the Maillard reaction. Exyl also produced reducing sugars by hydrolyzing AX and decreased the L* value through the Maillard reaction. However, approximately 2% of the AX content was relatively low in wheat flour and a limited amount of reducing sugar could be released by this enzyme reaction, and thus much less browning color was developed compared to an Aamyl reaction on starch that consists of 70–80% of wheat flour [40,41]. Between the untreated FSDB and FZDB controls, overall decreases in all the L*, a*, and b* values were significantly larger from FZDB when compared to FSDB; L* value decreased from 50.18 of FSDB to 42.80 of FZDB (Table 3). In the process of freezing and thawing of wheat dough, water exudation by syneresis concentrated the amount of wheat ingredients in dough, and the browning reaction might be facilitated, which in turn resulted in the darker color of bread crust. In addition, fermentation might not be complete due to the loss of yeast activity and unfermented sugars that remained, and thus the residual sugars possibly made a darker color due to the browning reaction during the baking process of frozen dough [42].

In this study, it was clearly shown that α-amylase treatment of bread dough resulted in the improvement of bread quality significantly. In addition, the endo-xyalanase action also partially assisted the prevention of quality loss in the beard crumb. Thus, positive effects of these enzymes on frozen bread dough can be promisingly utilized to prevent the quality loss of cold-chain distributed bread products. In terms of wheat cultivar development, the endogenous enzyme activities of wheat grains could be enhanced and thus the extra incorporation of exogenous enzymes might not be required to maintain bread quality.

## Figures and Tables

**Table 1:** Texture profile analysis results of enzyme-treated fresh and frozen bread doughs at storage of day 0 ^†^.

Enzyme Treatment Method	Hardness (N)		Cohesiveness		Gumminess (N)		Chewiness (J)	
	FSDB	FZDB	FSDB	FZDB	FSDB	FZDB	FSDB	FZDB
Untreated control	3.39 ± 0.09 ^a^	3.67 ± 0.35 ^a^	0.76 ± 0.02 ^b^	0.79 ± 0.02 ^a,b^	2.59 ± 0.12 ^b^	2.88 ± 0.26 ^a^	2.60 ± 0.12 ^a^	2.87 ± 0.26 ^a^
A20 ^‡^	1.84 ± 0.32 ^b^	1.80 ± 0.25 ^c^	0.86 ± 0.02 ^a^	0.77 ± 0.01 ^b,c^	1.57 ± 0.30 ^a^	1.39 ± 0.20 ^c^	1.58 ± 0.30 ^b^	1.39 ± 0.20 ^c^
A100	1.24 ± 0.46 ^b^	1.53 ± 0.26 ^c^	0.78 ± 0.03 ^b^	0.77 ± 0.01 ^b,c^	0.96 ± 0.32 ^a^	1.18 ± 0.19 ^c^	0.96 ± 0.33 ^b,c^	1.18 ± 0.19 ^c^
E20	1.81 ± 0.34 ^b^	2.68 ± 0.42 ^b^	0.80 ± 0.01 ^a,b^	0.81 ± 0.01 ^a^	1.44 ± 0.24 ^a^	2.18 ± 0.34 ^b^	1.44 ± 0.24 ^b,c^	2.18 ± 0.34 ^b^
E100	1.46 ± 0.31 ^b^	2.68 ± 0.62 ^b^	0.80 ± 0.01 ^a,b^	0.79 ± 0.02 ^a,b^	1.17 ± 0.23 ^a^	2.20 ± 0.34 ^b^	1.17 ± 0.23 ^b,c^	2.20 ± 0.33 ^b^
A20E20	1.83 ± 0.31 ^b^	1.65 ± 0.22 ^c^	0.81 ± 0.03 ^a,b^	0.79 ± 0.01 ^a,b^	1.46 ± 0.20 ^a^	1.30 ± 0.17 ^c^	1.46 ± 0.21 ^b,c^	1.30 ± 0.18 ^c^
A20E100	1.41 ± 0.19 ^b^	1.44 ± 0.08 ^c^	0.80 ± 0.01 ^a,b^	0.78 ± 0.02 ^a,b,c^	1.14 ± 0.16 ^a^	1.13 ± 0.05 ^c^	1.14 ± 0.17 ^b,c^	1.13 ± 0.05 ^c^
A100E20	1.42 ± 0.04 ^b^	1.32 ± 0.13 ^c^	0.78 ± 0.02 ^b^	0.78 ± 0.02 ^a,b,c^	1.11 ± 0.01 ^a^	1.03 ± 0.09 ^c^	1.11 ± 0.01 ^b,c^	1.03 ± 0.10 ^c^
A100E100	1.19 ± 0.24 ^b^	1.70 ± 0.35 ^c^	0.79 ± 0.03 ^a,b^	0.75 ± 0.01 ^c^	0.93 ± 0.16 ^a^	1.27 ± 0.25 ^c^	0.93 ± 0.17 ^c^	1.27 ± 0.26 ^c^

^†^ Means of triplicates. The values with different letters (^a,b,c^) in the same column are significantly different at *p* < 0.05. ^‡^ The values following the letters of A and E mean the level of each enzyme treatment in ppm as flour basis.

**Table 2:** Texture profile analysis results of enzyme-treated fresh and frozen bread doughs stored for 5 days ^†^.

	Hardness (N)		Cohesiveness		Gumminess (N)		Chewiness (J)	
	FS	FZ	FS	FZ	FS	FZ	FS	FZ
Untreated control	7.22 ± 0.09 ^a^	9.59 ± 0.12 ^a^	0.33 ± 0.02 ^a,b^	0.35 ± 0.01 ^b^	2.40 ± 0.15 ^a^	3.35 ± 0.15 ^a^	2.39 ± 0.15 ^a^	3.35 ± 0.15 ^a^
A20	5.59 ± 0.03 ^b,c^	6.86 ± 0.28 ^c^	0.33 ± 0.03 ^a,b^	0.35 ± 0.01 ^b^	1.84 ± 0.16 ^b,c^	2.41 ± 0.21 ^b,c,d^	1.85 ± 0.16 ^b,c^	2.41 ± 0.21 ^b,c,d^
A100	5.04 ± 0.31 ^c,d,e^	5.55 ± 0.36 ^e,f^	0.34 ± 0.04 ^a,b^	0.34 ± 0.01 ^b^	1.70 ± 0.28 ^b,c^	1.91 ± 0.16^d^	1.70 ± 0.28 ^b,c^	1.91 ± 0.16 ^d^
E20	6.32 ± 0.25 ^b^	9.45 ± 0.13 ^a^	0.33 ± 0.01 ^a,b^	0.35 ± 0.01 ^b^	2.10 ± 0.04 ^a,b^	3.34 ± 0.05 ^a^	2.10 ± 0.04 ^a,b^	3.34 ± 0.05 ^a^
E100	5.41 ± 0.21 ^c,d^	7.94 ± 0.06 ^b^	0.30 ± 0.02 ^b^	0.33 ± 0.03 ^b^	1.63 ± 0.11 ^b,c^	2.57 ± 0.24 ^b,c^	1.63 ± 0.12 ^c^	2.56 ± 0.24 ^b,c^
A20E20	5.48 ± 0.39 ^c,d^	6.30 ± 0.25 ^c,d^	0.35 ± 0.00 ^a,b^	0.33 ± 0.01 ^b^	1.91 ± 0.16 ^b,c^	2.10 ± 0.04 ^c,d^	1.91 ± 0.17 ^b,c^	2.10 ± 0.04 ^c,d^
A20E100	5.13 ± 0.40 ^c,d,e^	5.91 ± 0.30 ^d,e^	0.39 ± 0.01 ^a^	0.49 ± 0.04 ^a^	1.99 ± 0.14 ^a,b,c^	2.87 ± 0.05 ^a,b^	1.98 ± 0.15 ^a,b,c^	2.80 ± 0.18 ^a,b^
A100E20	4.71 ± 0.32 ^d,e^	5.53 ± 0.07 ^e,f^	0.36 ± 0.04 ^a,b^	0.46 ± 0.04 ^a^	1.69 ± 0.22 ^b,c^	2.56 ± 0.25 ^b,c^	1.69 ± 0.22 ^b,c^	2.56 ± 0.25 ^b,c^
A100E100	4.47 ± 0.27 ^e^	4.97 ± 0.22 ^f^	0.35 ± 0.02 ^a,b^	0.44 ± 0.05 ^a^	1.57 ± 0.07 ^c^	2.18 ± 0.34 ^c,d^	1.57 ± 0.07 ^c^	2.17 ± 0.34 ^c,d^

^†^ Means of triplicates. The values with different letters (^a,b,c,d,e,f^) in the same column are significantly different at *p* < 0.05.

**Table 3:** Effects of carbohydrate-active enzyme treatment on the crust color of breads ^†^.

	Fresh Dough Bread				Frozen Bread Dough			
	L*	A*	B*	ΔE	L*	A*	B*	ΔE
Untreated control	50.18 ± 0.61 ^a^	13.29 ± 0.15 ^a^	18.92 ± 0.10 ^b^	55.25	42.80 ± 1.01 ^a^	11.42 ± 0.82 ^a^	12.11 ± 1.72 ^a^	45.92
A20	46.35 ± 0.12 ^c^	12.68 ± 0.39 ^a,b^	14.92 ± 1.02 ^c,d^	50.32	39.41 ± 0.03 ^c^	10.31 ± 0.37 ^b^	9.42 ± 0.27 ^b^	41.81
A100	41.82 ± 0.46 ^f^	10.25 ± 0.13 ^c^	8.44 ± 0.18 ^f^	43.88	36.35 ± 0.19 ^d^	8.09 ± 0.62 ^c^	6.00 ± 0.42 ^c^	37.72
E20	49.77 ± 0.21 ^a^	12.98 ± 0.11 ^a^	21.90 ± 0.34 ^a^	55.90	41.14 ± 0.74 ^b^	10.81 ± 0.38 ^b^	9.18 ± 0.97 ^b^	43.52
E100	48.10 ± 0.21 ^b^	13.23 ± 0.12 ^a^	20.90 ± 0.11 ^a^	54.09	39.90 ± 0.12 ^b,c^	8.67 ± 0.55 ^c^	6.50 ± 0.46 ^c^	41.35
A20E20	44.55 ± 0.45 ^d^	12.94 ± 0.15 ^a^	15.74 ± 0.65 ^c^	48.99	39.61 ± 0.27 ^c^	8.30 ± 0.36 ^c^	5.94 ± 0.06 ^c^	40.90
A20E100	43.15 ± 0.18 ^e^	12.11 ± 0.16 ^b^	13.74 ± 0.07 ^d^	46.88	38.60 ± 0.23 ^c^	8.44 ± 0.35 ^c^	5.62 ± 0.30 ^c^	39.91
A100E20	40.96 ± 0.24 ^f^	10.51 ± 0.22 ^c^	10.77 ± 0.52 ^e^	43.64	36.56 ± 0.39 ^d^	4.94 ± 0.26 ^d^	2.52 ± 0.30 ^d^	36.98
A100E100	38.68 ± 0.61 ^g^	9.30 ± 0.32 ^d^	7.78 ± 0.62 ^f^	40.54	35.82 ± 0.04 ^d^	5.10 ± 0.22 ^d^	1.87 ± 0.07 ^d^	36.23

^†^ Means of triplicates. The values with different letters (^a,b,c,d,e,f,g^) in the same column are significantly different at *p* < 0.05.
